# HIV/AIDS health services in Manaus, Brazil: patient perception of quality and its influence on adherence to antiretroviral treatment

**DOI:** 10.1186/s12913-019-4062-9

**Published:** 2019-05-30

**Authors:** Carlued Leon, Tamar Koosed, Bryn Philibert, Cristina Raposo, Adele Schwartz Benzaken

**Affiliations:** 1 MANAUS, LLC, California, Los Angeles USA; 2 AIDS Healthcare Foundation Brazil, Sao Paulo, Brazil; 3 Foundation of Tropical Medicine Doctor Heitor Vieira Dourado, Manaus, Amazonas Brazil; 4 Global AIDS Healthcare Foundation, Los Angeles, California USA

**Keywords:** HIV services, Patient satisfaction, ART adherence, Decentralized healthcare, Brazil

## Abstract

**Background:**

Patient satisfaction is an important factor for both assessing the quality of healthcare and predicting positive health outcomes. This study assesses the influence of HIV/AIDS patients’ perception of the quality of health services on adherence to antiretroviral treatment using the decentralized care model in Manaus, Brazil.

**Methods:**

We conducted a non-randomized, cross-sectional analysis to explore the relationship between patient satisfaction and adherence to antiretroviral treatment (ART) in Manaus, Amazonas, Brazil. We also compared patient satisfaction levels at the city’s main hospital with those at smaller health units established to decentralize HIV/AIDS healthcare. Using survey responses from 812 patients and health data from 713 patients, we conducted descriptive and regression analyses to identify health center characteristics associated with higher patient satisfaction and higher adherence to treatment.

**Results:**

We found a clear and positive relationship between patient satisfaction with the quality of health services and adherence to ART. Patients who had better access to their health center and its services –mainly in the form of convenient location, shorter commute times, and shorter wait times— tended to rate the quality of services higher and were also more likely to adhere to ART. We also found higher levels of patient satisfaction and adherence to ART among patients served at decentralized health units than among patients served at the main hospital.

**Conclusions:**

The study’s results emphasize the importance of patients’ experience at the health center for improved health outcomes. While many of the factors that play a role in whether a patient adheres to ART or not are beyond the control of the health center, our findings highlight that health centers can importantly contribute to increased ART adherence by improving such experience. The study also showcases the potential benefits of decentralizing HIV care to increase patient satisfaction and, with it, adherence to ART.

**Electronic supplementary material:**

The online version of this article (10.1186/s12913-019-4062-9) contains supplementary material, which is available to authorized users.

## Background

Patient satisfaction with health services has been found to be an important factor in assessing the quality of healthcare. It has also been associated with increased adherence to treatment, utilization of health services, continuity of care, and even improved health conditions [1–3]. In the specific area of HIV/AIDS, the literature shows a strong relationship between patient satisfaction and patient care retention, adherence to antiretroviral therapy (ART), and viral load suppression [4–8]. Other studies have evaluated whether the decentralization of HIV/AIDS healthcare services has a positive effect on patient satisfaction. Here, the literature shows mixed results. Some studies find lower patient satisfaction among HIV/AIDS patients sent to decentralized units, while others find increased levels of patient satisfaction among HIV/AIDS patients routed to decentralized health centers [7–12]. This paper assesses the association between patient satisfaction and adherence to ART, along with differences between centralized and decentralized health units.

In an effort to improve the delivery of HIV/AIDS healthcare services in the city of Manaus, the capital city of the Brazilian state of Amazonas, the Brazilian Ministry of Health (MoH) developed a plan to decentralize HIV/AIDS patients from Manaus’ only reference hospital, the Fundação de Medicina Tropical (FMT), to several smaller specialized health units (SAEs for their acronym in Portuguese) with the capacity to provide HIV/AIDS care. As of 2016, 85% of the 9806 patients receiving ART in Manaus were treated at the main hospital, while the rest were treated at four decentralized health units.^1^

This study presents baseline results as part of a longer-term research project to assess the effectiveness of the decentralization effort in Manaus and to ensure the quality of services at the decentralized units receiving new patients does not deteriorate. While many factors influencing a patient’s adherence to ART are beyond the control of the health center, the results of this manuscript enlarge the available literature by identifying health center characteristics that can importantly contribute to increased ART adherence through enhanced patient satisfaction.

## Methods

We conducted a non-randomized, cross-sectional analysis of HIV/AIDS patients’ perceptions of the quality of health services in Manaus to assess the relationship between patient satisfaction and adherence to ART and its difference between centralized and decentralized health centers.

### Sampling Methods & Participants

The final sample included 812 HIV/AIDS patients: 410 patients interviewed at the central hospital and 402 patients interviewed at four decentralized health units. Both samples naturally picked up the 2:1 gender distribution of the epidemic in the state of Amazonas, with 528 men and 281 women participating in the study [13, 14].^2^ Samples were determined using a 95% confidence interval and additional participants were added to account for patient attrition or sample loss due to data quality issues. Table 1 shows the final sample distribution. Additional file 1 provides more details on the sample calculation.

**Table 1:** Final sample distribution

Proportion of participants by health center (*n* = 812)	Unweighted sample (%)	Weighted sample (%)
Central Hospital - FMT (*n* = 410)	50.49	85.44
Decentralized Health Units - SAEs (*n* = 402)	49.51	14.56

Study participants included men and women living with HIV/AIDS, aged 18 or older, who had at least one prior visit to the health centers. New patients were excluded from the study as the survey to determine satisfaction referred to the last consultation. Patients were selected during their routine consultations, prior to seeing the doctor, until the desired sample was reached. The demographic characteristics of the study participants are shown in Table 2.

**Table 2:** Demographic Characteristics of Study Participants

Characteristic	Total (*n* = 812)	Central Hospital (*n* = 410)	Decentralized Health Units (*n* = 402)	Chi-square *p*-value
Age (mean)	38.50	39.45	32.94	0.0001
Gender (%)				
Females	37.11	38.24	30.55	0.0000
Males	62.89	61.76	69.45	
Race (%)				
Black	5.75	5.85	5.17	0.2778
Mulatto	76.96	77.07	76.29	
White	12.86	12.44	15.31	
Asian	1.83	1.95	1.12	
Indigenous	2.60	2.68	2.11	
Education (%)				
Illiterate	0.50	0.49	0.58	0.3523
Incomplete primary education	23.38	24.39	17.48	
Complete primary education	7.71	8.05	5.75	
Incomplete secondary education	8.02	7.80	9.29	
Complete secondary education	38.26	38.05	39.50	
Incomplete tertiary education	9.75	9.27	12.59	
Complete tertiary education	9.98	9.51	12.74	
Graduate education	2.38	2.44	2.06	
Marital status (%)				
Never married	55.01	54.15	60.08	0.0037
Married	14.85	15.85	8.96	
Divorced/separated	3.21	3.17	3.45	
Widow(er)	4.00	4.39	1.73	
Lives with partner (but not married)	22.93	22.44	25.79	
Sexual Orientation (%)				
Heterosexual	67.64	70.17	52.82	0.0016
Homosexual	22.48	20.78	32.40	
Bisexual	9.88	9.05	14.77	
Employment (%)				
Employed	32.52	31.22	40.12	0.0032
Self-employed	16.50	16.59	15.97	
Unemployed with no source of income	27.05	25.61	35.50	
Unemployed with some financial support	14.98	16.34	0.7	
Retired	8.96	10.24	1.4	
Income (Brazilian Reais)				
Mean monthly income	1314.67	1315.85	1306.72	0.8987
Place of residence (%)				
Manaus	85.53	83.66	96.53	0.0000
Outside of Manaus	14.47	16.34	3.47	
Patient’s first CD4 count (%)	*n* = 713	*n* = 344	*n* = 369	
CD4 equal to 200/mm3 or greater	62.91	60.17	77.73	0.0240
CD4 less than 200/mm3	37.09	39.83	22.27	
First CD4 count (mean)	342.08	329.19	411.97	0.000
Viral load in last blood exam (%)	*n* = 678	*n* = 338	*n* = 340	
Viral load equal to 1000/mL or less	76.8	76.33	79.54	0.000
Viral load over 1000/mL	23.2	23.67	20.46	

### Research Instruments & Data Sources

We implemented a questionnaire through face-to-face interviews to measure overall patient satisfaction as well as satisfaction with specific aspects of health services. The instrument specifically gauged information about access to the health center, wait time, quality of communication with health professionals, type of exams conducted on the patient (e.g. lab exams, physical exams, etc.), provision of referrals to complementary services (i.e. financial, psychological, and/or alcohol/drug abuse support services), and general perceptions of quality of service. The questionnaire also collected information on patients’ socio-demographic characteristics, such as age, gender, education level, and employment, among others. The questionnaire was developed following the design of other validated tools, including the Health System Responsiveness section of the World Health Organization’s Health Survey, the Satisfaction with HIV/AIDS Treatment Interview Scale (SATIS), the Hospital Consumer Assessment of Healthcare Providers and Systems (HCAHPS), and the Patient Satisfaction Survey for HIV Ambulatory Care (PSS-HIV). Additional file 2 includes the final version of the questionnaire in English.

In addition to the questionnaire, we retrieved patients’ first CD4 count and latest viral load results from the National Disease Notification System (SINAN). Patients’ first CD4 count results were used as a proxy for timely diagnosis or timely initiation of ART, with a CD4 count equal to or greater than 200 copies/mm3 indicating that the patient was either timely diagnosed or had initiated ART in a timely manner [3]. Patients’ most recent available viral load results were used as a measure of adherence to ART, with a viral load equal to or less than 1000 copies/mL indicating that the patient adheres to ART. The data was collected between September and December 2016.

### Theoretical framework

In this paper, we aimed to identify health center characteristics associated with high patient satisfaction. Based on the empirical literature discussed above, we also sought to explore changes in adherence to treatment given high levels of satisfaction with the quality of care. To this end, we looked to answer two main research questions:

i) What are the most relevant health center-related factors associated with high levels of patient satisfaction? This question was explored using model specification: *Patient Satisfaction = F(health center characteristics, patient characteristics)*
ii) Does high patient satisfaction lead to higher levels of adherence to ART? This question was answered using model specification: *Adherence to ART = F(patient satisfaction, patient characteristics).*

### Data analysis

We conducted descriptive and regression analyses to identify patient and health center characteristics that influence patient satisfaction, as well as to assess the correlation between patients’ perceptions on the quality of care and adherence to ART. Pearson’s chi-squared tests were used to check for the statistical significance of descriptive results. Logistic regression analysis was used to identify health center characteristics associated with high patient satisfaction and to determine the likelihood of adherence to treatment given high patient satisfaction with the quality of care. Because patient satisfaction with health services and adherence to ART can be affected by health facility factors as well as individual level factors, we explored the use of multi-level logistic models. In the case of adherence to treatment, the likelihood ratio test indicated that a multi-level model was a better fit than the single-level model and it was used accordingly. The analysis was done using information from all patients as well as by comparing results between the two types of health units, centralized versus decentralized. We employed sampling weights to ensure that results reflect the real distribution of patients across the five health centers (Table 1).

The regression models for patient satisfaction included the following predictors: i) *commute time to the health center*, as a long commute to the health facility has been associated with lower patient satisfaction [1, 15, 16]; ii) *wait time*, as patients who experience long wait times to see a health professional are less likely to be satisfied with the quality of health service [5, 6, 15, 17]; iii) *convenience of health center’s location,*^3^ which refers to the patient’s perception of the location of the health center and not its actual location (e.g. health facility is not close to patient’s home, but it is on the way to work) and has been associated with higher levels of patient satisfaction [5, 16]; iv) *respectful treatment from nurses*, since nurses tend to be the health professionals to interact the most with the patients and the quality of communication with them have been found to influence satisfaction levels [6, 15]; and v) *respectful treatment from doctors*, as patients who report feeling respected by the doctor also report higher levels of satisfaction with services [6, 17]. In addition, the models controlled for the following personal characteristics: i) *age*, as older patients tend to report higher satisfaction with services [2, 5]; ii) *gender*, as females tend to report higher patient satisfaction than male patients [5, 6, 16]; iii) *education*, as higher levels of education have been associated with higher satisfaction [2, 6, 18]; iv) *race*, since specific racial groups face more discrimination and challenges to access health services, making them more likely to report lower patient satisfaction [19]; v) *sexual orientation*, as heterosexual patients tend to report lower levels of patient satisfaction than individuals of other sexual identity [20]; vi) *income level*, as richer patients tend to report lower levels of satisfaction than poorer individuals [2, 5]; vii) *place of residence*, as patients living in communities with little to no access to specialized HIV/AIDS tend to be more satisfied with any health services they can access than patients in areas with more options for specialized healthcare [9]; and viii) *type of health center* (centralized versus decentralized), as patients served in decentralized health facilities tend to report higher patient satisfaction [9, 21]. Regression models were conducted in sequence, whereby each predictor was added one at a time to observe changes in patient satisfaction until a final model was achieved with the health center characteristics that best explain variations in patient satisfaction.

In regard to the effect of patience satisfaction on adherence to ART, the regression models included *general patient satisfaction* as the main explanatory variable. These models controlled for the following patient characteristics: i) *age*, as older patients are more likely to adhere to treatment than younger individuals [22, 23]; ii) *gender*, since adherence to ART tends to be lower among female patients than male patients [22, 24, 25]; iii) *education*, as patients with more levels of education are more likely to adhere to treatment than those with no or lower education levels [24, 25]; iv) *race*, as racial groups traditionally discriminated against and with difficult access to health services have been found to adhere less to treatment [26–28]; v) *sexual orientation*, as treatment continuation tends to be lower among heterosexual patients than among patients of other sexual identity [29–31]; vi) *income level*, since richer patients tend to adhere better to ART than poorer patients [22]; vii) *place of residence*, as patients in communities with little to no access to HIV/AIDS health services tend to show higher adherence to treatment [32]; and viii) *type of health center* (centralized versus decentralized), as patients in decentralized health facilities tend to adhere better to ART than patients in centralized health units [21, 32].

## Results

We found a clear and positive relationship between patient satisfaction with the quality of health services and adherence to ART. Patients who had better access to their health center and its services tended to rate the quality of services higher and were also more likely to adhere to ART. Results also show that patient satisfaction and adherence to ART were higher among patients at decentralized health units.

### Patient satisfaction & access to health services

Almost 82% of all interviewed patients rated the quality of services as ‘excellent’ or ‘good’ (Table 3). However, when disaggregating results between centralized and decentralized health units, we observed higher satisfaction among patients at decentralized facilities (Central Hospital: 81%; Decentralized Health Units: 86%; *p*-value 0.0003). Differences in satisfaction levels between the two types of health units are more pronounced when looking at the highest satisfaction category, “excellent” (Central Hospital: 19.51%; Decentralized Health Units: 35.59%; *p*-value: 0.0172).

In regard to access to health services, patients at decentralized facilities were nearly twice as likely to report a commute time of less than 30 min than patients at the centralized unit (Central Hospital: 19.02%; Decentralized Health Units: 34.25%; *p*-value: 0.0182). Patients at decentralized units were also almost three times more likely to report wait times to see a health professional under 30 min (Central Hospital: 8.05%; Decentralized Health Unit: 23.54%; *p*-value: 0.0015), whereas patients at the main hospital were more likely to report wait times of one hour or more (Central Hospital: 67.55%; Decentralized Health Units: 40.67%; *p*-value: 0.001). Almost all patients at decentralized care facilities reported waiting less than 30 min to retrieve their ART medication, compared to only a third of patients at the central hospital who reported the same (Central Hospital: 32.98%; Decentralized Health Units: 92.83%; *p*-value: 0.0018). The fastest patients at the main hospital could find an available appointment was, on average, 70 days, compared to an average of 31 days for patients at decentralized units (Central Hospital: 69.50 days; Decentralized Health Units: 31.28 days; *p*-value: < 0.001).

As for the quality of communication with health professionals, patients at decentralized facilities were more likely to report respectful treatment from nurses (Central Hospital: 77.80%; Decentralized Health Units: 92.04%; *p*-value: 0.0184), that nurses provided information about their health or treatment in a simple and clear way (Central Hospital: 73.90%; Decentralized Health Units: 93.79%; *p*-value: 0.0092), and that nurses generally answer their questions (Central Hospital: 58.29%; Decentralized Health Units: 83.71%; p-value: 0.0063). Similarly, patients at decentralized health centers were more likely to report that doctors provided information about their health or treatment in a simple and clear way (Central Hospital: 92.68%; Decentralized Health Centers: 97.15%; p-value: 0.0160) and that doctors generally answer their questions (Central Hospital: 82.44%; Decentralized Health Units: 94.76%; *p*-value: 0.0276).

**Table 3:** General patient satisfaction and accessibility indicators

	Total (*n* = 812)	Central Hospital (*n*= 410)	Decentralized Health Units (*n*= 402)	Chi-square *p*-value
General satisfaction with quality of health services (%)				
Excellent quality	21.85	19.51	35.59	0.0172
Good quality	59.68	61.22	50.68	
Average quality	15.83	16.59	11.43	
Bad quality	2.10	2.20	1.53	
Terrible quality	0.53	0.49	0.78	
Commute time to health center (%)				
Less than 30 min	21.24	19.02	34.25	0.0182
30 min to 1 h	33.46	32.20	40.88	
1–2 h	31.02	32.93	19.87	
2–3 h	6.50	7.07	3.11	
More than 3 h	7.57	8.54	1.89	
Don’t know/No response	0.21	0.24	–	
Wait time (%)				
Less than 30 min	10.31	8.05	23.54	0.0015
30 min to 1 h	25.97	24.39	35.22	
1–2 h	23.05	23.41	20.92	
2–3 h	18.99	20.73	8.79	
More than 3 h	21.60	23.41	10.96	
Don’t know/no response	0.08	–	0.56	
Time to reschedule a missed appointment (# of days)				
Average number of days to reschedule	63.93	69.50	31.28	< 0.0001
Time patient waited at pharmacy to be helped last time he/she went to pick up HIV medication (%)				
30 min or less	43.97	32.98	92.83	0.0018
30 min to 1 hour	27.88	32.98	5.18	
1–2 h	20.22	24.61	0.73	
2–3 h	5.13	6.28	–	
More than 3 h	1.71	2.09	–	
Don’t remember/no response	1.09	1.05	1.26	
Communication with nurses (%)				
Respectful treatment from nurses (yes)	79.88	77.80	92.04	0.0184
Nurses provided information about patient’s health or treatment in a simple and clear way (yes)	76.80	73.90	93.79	0.0092
Nurses answered patient’s questions (yes)	61.99	58.29	83.71	0.0063
Communication with doctors (%)				
Respectful treatment from doctors (yes)	95.34	94.88	98.02	0.1069
Doctors provided information about patient’s health or treatment in a simple and clear way (yes)	93.33	92.68	97.15	0.0160
Doctor answered patient’s questions (yes)	84.23	82.44	94.76	0.0276

When exploring the association between patient satisfaction and all indicators of health services accessibility, health center location, wait time, and interactions with nurses were among the most prominent factors influencing patient satisfaction (Table 4). Patients who believed their health center was conveniently located^3^ were three times more like to be satisfied with its healthcare services than patients who thought otherwise (adjusted odds ratio (aOR): 2.70; 95% confidence interval (CI): 2.18–3.35; *p*-value < 0.0001). Patients who generally waited more than three hours to see a doctor were 60% less likely to be satisfied with the services (aOR: 0.40; 95% CI: 0.31–0.51; *p*-value < 0.0001). Patients who said nurses usually treat them with respect were 50% more likely to be satisfied (aOR: 1.50; 95% CI: 1.03–2.20; *p*-value: 0.040). Please refer to Additional file 3 for further details.

At the central hospital, patient satisfaction is associated with commute time and health center location (Table 5), whereas patient satisfaction at the decentralized health units is driven by shorter wait times and quality of communication with nurses (Table 6). At the main hospital, patients who reported a commute time over one hour were 52% less likely to be satisfied with health services (aOR: 0.48; 95% CI: 0.23–0.98; *p*-value: 0.044), while patients who said the health center is conveniently located were nearly three times more likely to be satisfied (aOR: 2.49; 95% CI: 1.07–5.77; *p*-value: 0.034). At the decentralized health centers, patients who reported wait times to see a doctor above one hour were less likely to be satisfied (30 min-1 h wait: aOR: 0.51, 95% CI: 0.29–0.92, p-value: 0.033; 1–2 h wait: aOR: 0.24, 95% CI: 0.18–0.32, p-value < 0.001; 2–3 h wait: aOR: 0.32, 95% CI: 0.21–0.50, p-value: 0.002; more than 3-h wait: aOR: 0.08, 95% CI: 0.05–0.12, p-value < 0.001). Patients at decentralized units who said nurses generally treat them with respect were nearly four times more likely to be satisfied with health services than patients who reported otherwise (aOR: 3.64, 95% CI: 2.48–5.35, p-value: 0.001). Please refer to Additional files 4 and 5 for further details.

**Table 4:** Factors associated with high patient satisfaction

Factors associated with high patient satisfaction at centralized and decentralized health units (*n* = 810)	Adjusted OR	95% CI		*p*-value
Health center’s location				
Health center is conveniently located	2.70	2.18	3.35	0.000
Otherwise	1			
Wait time				
Less than 30 min	1			
30 min to 1 h	0.58	0.12	2.83	0.413
1–2 h	0.52	0.13	2.11	0.282
2–3 h	0.45	0.08	2.46	0.279
More than 3 h	0.40	0.31	0.51	0.000
Respectful treatment from nurses				
No	1			
Yes	1.50	1.03	2.20	0.040

*OR* Odds Ratios. Adjusted OR control for age, gender, education, race, sexual orientation, income, place of residence (Manaus vs. elsewhere), and/or health center

**Table 5:** Factors associated with high patient satisfaction at central hospital

Factors associated with high patient satisfaction at the central hospital only (*n* = 409)	Adjusted OR	95% CI		*p*-value
Commute time to health center				
Less than 30 min	1			
30 min to 1 h	0.55	0.28	1.12	0.099
More than 1 h	0.48	0.23	0.98	0.044
Health center’s location				
Health center is conveniently located	2.49	1.07	5.77	0.034
Otherwise	1			
Correlation between commute time and convenience of health center’s location	Coefficient			
Health center’s location	1			
Commute time to health center	0.1051			<0.05

*OR* Odds Ratios. Adjusted OR control for age, gender, education, race, sexual orientation, income, place of residence (Manaus vs. elsewhere), and/or health center

**Table 6:** Factors associated with high patient satisfaction at decentralized health units

Factors associated with high patient satisfaction at decentralized health units only (*n* = 400)	Adjusted OR	95% CI		*p*-value
Waiting time				
Less than 30 min	1			
30 min to 1 h	0.51	0.29	0.92	0.033
1–2 h	0.24	0.18	0.32	0.000
2–3 h	0.32	0.21	0.50	0.002
More than 3 h	0.08	0.05	0.12	0.000
Respectful treatment from nurses				
No	1			
Yes	3.64	2.48	5.35	0.001

*OR* Odds Ratios. Adjusted OR control for age, gender, education, race, sexual orientation, income, place of residence (Manaus vs. elsewhere), and/or health center

### Patient satisfaction & adherence to ART

Using patients’ latest available viral load count as a proxy for adherence to ART, we found that high patient satisfaction increases the likelihood of adhering to ART (Table 7). For the purpose of this analysis, patient satisfaction was considered as high when patients rated their general satisfaction with the quality of health services as excellent. We also assumed that patients receiving ART for at least one year and with viral load less than 1000 copies/mL adhered to ART. After controlling for several patient and health center characteristics, patients who rated the overall quality of services as “excellent” were nearly two times more likely to adhere to ART than patients who were less satisfied or not satisfied at all with the services (aOR: 1.99, 95% CI: 1.02–3.89, *p*-value: 0.043).

Results also show that adherence to treatment is higher at decentralized health units than at the central hospital. Nearly 80% of patients at decentralized facilities had a viral load under 1000 copies/mL, compared to 76% of patients at the main hospital with the same results (Central Hospital: 76.33%; Decentralized Health Units: 79.54%; *p*-value < 0.001). Please refer to Additional file 6 for further details.

**Table 7:** Effect of patient satisfaction on adherence to ART

Effect of patient satisfaction on adherence to ART (*n* = 499)	Adjusted OR	95% CI		*p*-value
Patient satisfaction				
Very satisfied	1.99	1.02	3.89	0.043
Otherwise	1			

*OR* Odds Ratios. Adjusted OR control for age, gender, education, race, sexual orientation, income, place of residence (Manaus vs. elsewhere), and/or health center. Adherence to ART is measured through viral load. A Viral load less than 1000 copies/mL indicates patient adheres to ART

### Timely diagnosis and initiation of ART

Using patients’ first CD4 count as a measure of timely diagnosis and/or timely initiation of ART, we found that patients at decentralized units tend to be diagnosed and initiate ART more timely than patients at the centralized health unit (Table 8). For the purpose of this analysis, we assumed that patients with a CD4 count equal or greater than 200 copies/mm3 were timely diagnosed or initiated ART timely [3]. A larger proportion of patients at decentralized facilities were timely diagnosed or timely initiated ART when compared to patients at the main hospital (Central Hospital: 60.17%; Decentralized Health Units: 77.73%; *p*-value: 0.024). In regression analysis, we found that when patients were served at the decentralized health units, they were twice as likely to have been diagnosed or have initiated ART timely than patients served at the central hospital (cOR: 2.31; 95% CI: 1.16–4.62; *p*-value: 0.027).

**Table 8:** Effect of type of health center on timely diagnosis/timely ART initiation

Effect of type of health center (centralized vs. decentralized) on timely diagnosis/timely ART initiation (n = 713)	Crude OR	95% CI		*p*-value
Health center				
Central Hospital (FMT)	1			
Decentralized Health Units (SAEs)	2.31	1.16	4.62	0.027

*OR* Odds Ratios. Crude OR do not control for other variables. Timely diagnosis/timely ART initiation of ART is measured through patients’ first CD4 count. A CD4 count equal to or greater than 200 copies/mm3 indicate timely diagnosis/ART initiation

## Discussion

The results of this study confirm the positive association between patient satisfaction with quality of care and adherence to ART. As found in other studies [4, 7, 8, 11], patients who are satisfied with the health services at the center where they receive treatment are also more likely to adhere to ART. This implies that improving the care experience of HIV/AIDS patients could be an innovative approach to advancing HIV outcomes.

Patients at both centralized and decentralized health units generally reported high levels of patient satisfaction. However, as observed in other studies [6, 15, 33–35], patients reported much lower levels of satisfaction with specific aspects of health services, including health center location, wait times to see a health professional, and wait times to retrieve ART medication from the health center’s pharmacy.

The main factors positively affecting patient satisfaction were accessibility to the health center —in the form of convenient location, shorter wait times, and shorter commute times— and quality of communication with health staff —specifically, positive interactions with the nurses. Our findings on wait times are consistent with those of other studies showing wait times to be the strongest determinants of patient satisfaction [6, 15, 36, 37]. In regard to communication with health professionals, other studies have also found the quality of communication with health staff to affect patient satisfaction [18] and adherence to treatment [38, 39].

In regard to the relationship between the decentralization of HIV/AIDS care and patient satisfaction, the literature shows mixed results. Some studies have found higher levels of satisfaction at decentralized health units [7, 8, 10, 11], while others reported negative effects on patient satisfaction [9, 12]. In our study, we found higher levels of patient satisfaction and of adherence to ART among patients served at decentralized health centers. These results likely respond to differences in the location and volume of patients between the central hospital and the decentralized health units. FMT, the central hospital, is located in the middle of the city, with more traffic around it, whereas the decentralized health units are located in semi-urban areas that are generally more accessible. In addition, the proportion of patients who live outside of Manaus and are served at the central hospital is higher than that of non-locals receiving services at decentralized health units. The overconcentration of patients at the central hospital (85% of all patients receiving ART in the city are served at this hospital) likely prevents health professionals from providing a more dedicated service to patients, affecting patients’ perceptions around the quality of communication with health staff. Differences in wait times to schedule appointments, to see a doctor, and to retrieve medications may also contribute to the observed differences in overall satisfaction between centralized and decentralized health centers.

The study’s results are comprehensive in their understanding of health center characteristics that contribute to satisfaction for patients that routinely utilize the services at the centers. However, there are a number of potential limitations to this study. We only interviewed patients who were present for their appointments and we did not control for whether interviewed patients had a consistent history of check-up attendance. It is possible that, for patients who missed their appointment or who do not go for check-ups regularly, the characteristics that determine patient satisfaction and ART adherence are different than the ones reported in this study. In addition, the study used viral load under 1000 copies/mL as a proxy for treatment adherence, but some patients may adhere to treatment and still show a viral load above 1000 copies/mL due to factors other than adherence, such as drug resistance, which the study does not control for. At the time of the study, the first line regime for most patients was Efavirenz-based, for which the Ministry of Health estimated a national resistance prevalence of 5.62% in 2016 [40, 41]. In this sense, the study may be underestimating the levels of ART adherence at the studied health facilities as well as the effect of patient satisfaction on adherence to treatment. As the surveys were administered at the health centers, patients may have been less inclined to express any negative feelings about health unit or the health staff. Likewise, patients who declined to participate in the study may have had negative opinions about the heath services. These limitations may have introduced an upward bias into the reported results.

## Conclusion

The study’s results emphasize the importance of patients’ experience at the health center for improved health outcomes. While many factors play a role in whether a patient adheres to ART or not, some beyond the control of the health center, these findings highlight that health centers can importantly contribute to increased ART adherence by improving such experience. The study also showcases the potential benefits of decentralizing HIV care to increase patient satisfaction and, with it, adherence to ART.

## Additional files

Additional file 1: Sample Size Calculations. This file describes the formula used to estimate the study sample. (PDF 61 kb)

Additional file 2: Study Questionnaire. This file includes the final version of the questionnaire used for this study in English. (PDF 155 kb)

Additional file 3: General Factors Associated with Patient Satisfaction. This file presents regression results on factors associated with patient satisfaction at both the central hospital and the decentralized health units. (PDF 57 kb)

Additional file 4: Factors Associated with Patient Satisfaction at Central Hospital. This file presents regression results on factors associated with patient satisfaction at the central hospital. (PDF 55 kb)

Additional file 5: Factors Associated with Patient Satisfaction at Decentralized Health Units. This file presents regression results on factors associated with patient satisfaction at the decentralized health units. (PDF 57 kb)

Additional file 6: Effect of Patient Satisfaction on Adherence to ART. This file presents regression results on the effect of patient satisfaction on adherence to treatment at both the central hospital and the decentralized health units. (PDF 56 kb)

## Abbreviations

AIDS: Acquired Immunodeficiency Syndrome
aOR: Adjusted Odds Ratio
ART: Antiretroviral Therapy
CI: Confidence Interval
cOR: Crude Odds Ratio
FMT: Fundação de Medicina Tropical (Tropical Medicine Foundation)
HIV: Human Immunodeficiency Virus
IRB: Institutional Review Board
MoH: Ministry of Health
SAE: Serviços de Atenção Especializada (Specialized Health Units)
SINAN: National Disease Notification System (Brazil)

## Footnotes

1 Data extracted from databases Sistema de Controle Logístico de Medicamentos (SICLOM) and Sistema de Controle de Exames Laboratoriais (SISCEL).

2 Three patients declined to disclose their gender

3 Convenience of health center’s location is related to patient’s perception of the location of the health center and not to the actual location of the health center (distance or commute time). Convenience may not necessarily respond to distance or commute time. For example, a health center may not be close to the patient’s home, but it can be convenient if it is on the way between home and work, if there is good public transportation to the health center, or if it is far enough from his/her neighborhood so as to keep the HIV positive status private from others in his/her community.
