# Supplementary material for: HIV/AIDS health services in Manaus, Brazil: patient perception of quality and its influence on adherence to antiretroviral treatment
Source: BMC Health Serv Res. 2019 May 30;19:344. doi: 10.1186/s12913-019-4062-9 (PMC6543648; doi:10.1186/s12913-019-4062-9)
Supplement: Supplementary file 1 — Sample Size Calculations. This file describes the formula used to estimate the study sample. (PDF 61 kb) [file 12913_2019_4062_MOESM1_ESM.pdf]

Sample size calculation

The study drew a statistically proportional sample of the population of interest: patients living with HIV/AIDS who are registered to receive care at the central hospital (FMT) and the four decentralized health units (SAEs). The formula used to calculate the sample size is:

$$\frac{\frac{z^2 \times p\,(1-p)}{e^2}}{1 + \left(\frac{z^2 \times p\,(1-p)}{e^2 N}\right)}$$

where:
N = population size
z = z-score
e = margin error

Even though the majority of patients were concentrated at the main hospital (FMT), the two samples were equated since the patient population at the decentralized health units is expected to increase as a result of the decentralization process. Because the number of patients that will be decentralized was unknown at the time of this baseline study, this equalization of samples will allow the research team to compare the two groups after the decentralization without losing statistical power. The table below shows the sampling strategy and compares the desired sample with the actual sample collected:

**Unit**	**Population** (No. of patients registered at FMT and SAEs in Manaus)	**Sample** (minimum sample statistically representative at 95% CI)	**Desired Sample** (including oversampling for attrition)	**Actual Sample Collected**
Central Hospital (FMT)	8,378	368	400	410
Decentralized Health Units (SAE)	1,428	303	400	402
