# Supplementary material for: HIV/AIDS health services in Manaus, Brazil: patient perception of quality and its influence on adherence to antiretroviral treatment
Source: BMC Health Serv Res. 2019 May 30;19:344. doi: 10.1186/s12913-019-4062-9 (PMC6543648; doi:10.1186/s12913-019-4062-9)
Supplement: Supplementary file 2 — Study Questionnaire. This file includes the final version of the questionnaire used for this study in English. (PDF 155 kb) [file 12913_2019_4062_MOESM2_ESM.pdf]

Final Questionnaire (English)

This questionnaire was developed for the study "HIV/AIDS Health Services in Manaus, Brazil: Patient Perception of Quality and its Influence on Adherence to Antiretroviral Treatment" (BHSR-D-18-01154). It was administrated in Portuguese using mobile technology. It was translated into English for publication purposes.

INTRODUCTION

My name is _________ and I work for AHF. AHF is a non-profit organization based in the United States and conducts activities to address the HIV/AIDS epidemic. AHF began activities in Brazil in 2013 to support actions to combat HIV/AIDS in the country, in partnership with the Ministry of Health and civil society. I would like to ask you a few questions about the quality of medical care you receive at this health unit. Your answers will help us understand what works well, what does not work, and what needs to improve. Your participation is voluntary and your responses confidential. Regardless of whether or not you participate in this research, the services you receive at this health unit will not be affected. Could we start the interview?

- Yes (1)
- No (2) If this response, jump to 79

1. [INTERVIEWER: Please selected you Interviewer Code]

- P1 (1)
- P2 (2)
- P3 (3)
- P4 (4)
- P5 (5)
- P6 (6)

2. [INTERVIEWER: Did the participant sign the Terms of Free and Clarified Consent (Termos de Consentimento Livre e Esclarecido, TCLE)?]

- Yes (1)
- No (0) If this response, jump to 79

3. [INTERVIEWER: Select the health center where this interview is taking place]

- Fundação de Medicina Tropical Doutor Heitor Vieira Dourada (FMT) (1)
- SAE Dr. Comte Telles (2)
- SAE Dr. José Antônio da Silva/PMO (3)
- SAE Dr. Franco de Sá (4)
- SAE Dr. Antônio Reis (5)
- UBS Arthur Virgílio Filho (6)
- UBS Theodomiro Garrido (7)
- UBS Leonor de Freitas (8)
- UBS Maria Leonor Brilhante (9)

4. When was your last visit to this health unit to follow up on your HIV/AIDS treatment? [INTERVIEWER: Select the most appropriate option based on participant's answer]

- Patient's very first visit for HIV/AIDS treatment (0) If this response, jump to 79
- Less than a month ago (1)
- More than a month but less than 6 months ago (2)
- More than 6 months but less than 1 year (3)
- More than 1 year ago (4)
- Patient dropped out of treatment and this is the first visit to resume treatment (55) If this response, jump to 79
- [Doesn't want to answer] (99)

5. How long have you been receiving HIV/AIDS treatment at this health unit? [INTERVIEWER: Select the most appropriate option based on participant's answer]

- Less than a year ago (1)
- 1-2 years ago (2)

- 2-5 years ago (3)
- More than 5 years ago (4)
- [Doesn't want to answer] (99)

6. I'm going to ask you some questions about your access to healthcare. All the questions I'm going to ask now are about your last visit to this health unit.

7. If you missed an appointment at this health unit, in how many DAYS would you be able to get another appointment? [INTERVIEWER: If patient responds in weeks or months, calculate the corresponding number of days. 1 week = 7 days; 1 month = 30 days. Write "MMM" if user does not want to respond]

________________ DAYS

8. For your last consultation, how long did it take you to get from your home to the health unit? [**INTERVIEWER**: **Select the most appropriate option based on participant's answer**]

- Less than 30 minutes (1)
- Between 30 minutes and 1 hour (2)
- More than 1 hour and less than 2 hours (3)
- More than 2 hours and less than 3 hours (4)
- More than 3 hours (5)
- [Doesn't know/doesn't remember] (88)
- [Doesn't want to answer] (99)

9. How would you rate the location of this health unit? [INTERVIEWER: Read answer options out loud]

- Very convenient (1)
- Convenient (2)
- More or less convenient (3)
- Inconvenient (4)
- Very inconvenient (5)
- [Doesn't want to answer] (99)

10. Thinking about your last visit, how long did you wait to see a doctor or a nurse? [**INTERVIEWER**: **Select the most appropriate option based on participant's answer**]

- Less than 30 minutes (1)
- Between 30 minutes and 1 hour (2)
- More than 1 hour and less than 2 hours (3)
- More than 2 hours and less than 3 hours (4)
- More than 3 hours (5)
- [Doesn't know/doesn't remember] (88)
- [Doesn't want to answer] (99)

11. How would you rate the wait time to be seen by a doctor or a nurse in your last visit to this health unit? [INTERVIEWER: Read answer options out loud]

- Very good (1)
- Good (2)
- Reasonable (3)
- Bad (4)
- Very bad (5)
- [Doesn't want to answer] (99)

12. How often do you come to your appointment and you do not receive the expected healthcare services because the doctor did not show up? [INTERVIEWER: Read answer options out loud]

- Always (1)
- Frequently (2)
- Sometimes (3)

- Rarely (4)
- Never (5)
- [Doesn't want to answer] (99)

13. I'm now going to ask you some questions about your communication with the health professionals at this health unit. By health professionals we mean nurses and doctors.

14. During your last consultation, did the NURSES treat you with kindness and respect?

- Yes (1)
- No (0)
- [Doesn't know/doesn't remember] (88)
- [Doesn't want to answer] (99)

15. During your last consultation, did the NURSES give you information about your consultation, your health or about your treatment in a way that was simple and easy to understand?

- Yes (1)
- No (0)
- [Doesn't know/doesn't remember] (88)
- [Doesn't want to answer] (99)

16. During your last consultation, did the NURSES answer all the questions you had?

- Yes (1)
- No (0)
- I did not have/ask questions (2)
- [Doesn't know/doesn't remember] (88)
- [Doesn't want to answer] (99)

17. During your last consultation, did the DOCTORS treat you with kindness and respect?

- Yes (1)
- No (0)
- [Doesn't know/doesn't remember] (88)
- [Doesn't want to answer] (99)

18. During your last consultation, did the DOCTORS give you information about your consultation, your health or about your treatment in a way that was simple and easy to understand?

- Yes (1)
- No (0)
- [Doesn't know/doesn't remember] (88)
- [Doesn't want to answer] (99)

19. During your last consultation, did the DOCTORS answer all the questions you had?

- Yes (1)
- No (0)
- I did not have/ask questions (2)
- [Doesn't know/doesn't remember] (88)
- [Doesn't want to answer] (99)

20. During your last visit, when someone in your family or someone you know wanted to talk to the DOCTORS, were they allowed? [INTERVIEWER: Read answer options out loud]

- Yes (1)
- No, they could not talk to the doctors (2) If this response, jump to 22
- No relatives or friends were present (3) If this response, jump to 22
- None of my relatives or friends wanted to talk to the doctors (4) If this response, jump to 22

- I did not want my relatives or friends to talk to the doctors (5) If this response, jump to 22
- [Doesn't know/doesn't remember] (88) If this response, jump to 22
- [Doesn't want to answer] (99) If this response, jump to 22

21. During your last visit, did the nurses or the doctors give your family or someone you know all the information they needed to help you with your treatment? [INTERVIEWER: Read answer options out loud]

- Yes (1)
- No, they did not receive any information (2)
- No relatives or friends were present (3)
- None of my relatives or friends needed information (4)
- I did not want my relatives or friends to talk to the nurses or doctors (5)
- [Doesn't know/doesn't remember] (88)
- [Doesn't want to answer] (99)

22. Sometimes the doctor says one thing and the nurse says another, giving different information to the patient. Did this happen during your last consultation?

- Yes (1)
- No (0)
- [Doesn't know/doesn't remember] (88)
- [Doesn't want to answer] (99)

23. I'm now going to ask you some questions about the medical attention received in your last visit to this health center.

24. During your last consultation, did you have any pain?

- Yes (1)
- No (0) If this response, jump to 26
- [Doesn't know/doesn't remember] (88) If this response, jump to 26
- [Doesn't want to answer] (99) If this response, jump to 26

25. Did the nurses or the doctors did everything they could to ameliorate your pain?

- Yes (1)
- No (0)
- [Doesn't know/doesn't remember] (88)
- [Doesn't want to answer] (99)

26. During your last consultation, did health professionals explain the purpose of your medication in a way that was simple and easy to understand? [INTERVIEWER: Read answer options out loud]

- Yes (1)
- No (0) If this response, jump to 28
- I didn't need explanation (2) If this response, jump to 28
- I do not need medication (3) If this response, jump to 29
- [Doesn't know/doesn't remember] (88) If this response, jump to 28
- [Doesn't want to answer] (99) If this response, jump to 28

27. During your last consultation, did health professionals explain the side effects of your medications in a way that was simple and easy to understand? [INTERVIEWER: Read answer options out loud]

- Yes (1)
- No (0)
- I didn't need explanation (2)
- [Doesn't know/doesn't remember] (88)
- [Doesn't want to answer] (99)

28. From the options I am going to read, which one best describes your access to the medication for your HIV/AIDS treatment? [INTERVIEWER: Read answer options out loud]

- I always find the medication (1)
- I find the medication most of the time (2)
- Sometimes I find the medication (3)
- I rarely find the medication (4)
- I never find the medication (5)
- [Doesn't know/doesn't remember] (88)
- [Doesn't want to answer] (99)

29. During your last consultation, did health professionals explain what medical exams you needed to do and how often you needed to do those exams?

- Yes (1)
- No (0)
- [Doesn't know/doesn't remember] (88)
- [Doesn't want to answer] (99)

30. During your last consultation, did health professionals explain the risks associated with HIV/AIDS and how you can prevent new infections and complications?

- Yes (1)
- No (0)
- [Doesn't know/doesn't remember] (88)
- [Doesn't want to answer] (99)

31. During your last consultation, did health professionals explain how you could avoid infecting others with HIV?

- Yes (1)
- No (0)
- [Doesn't know/doesn't remember] (88)
- [Doesn't want to answer] (99)

32. In the last 12 months, did you get any physical examination done; for example, did healthcare staff measure your blood pressure, weight, height, etc.?

- Yes (1)
- No (0)
- [Doesn't know/doesn't remember] (88)
- [Doesn't want to answer] (99)

33. In the last 12 months, have you done any laboratory tests, such as blood tests, stool tests, CD4+, viral load, etc.?

- Yes (1)
- No (0)
- [Doesn't know/doesn't remember] (88)
- [Doesn't want to answer] (99)

34. In the last 12 months, did you take any tuberculosis tests?

- Yes (1)
- No (0) If this response, jump to 36
- [Doesn't know/doesn't remember] (88) If this response, jump to 36
- [Doesn't want to answer] (99) If this response, jump to 36

35. Have you ever been or are you being treated for tuberculosis? [INTERVIEWER: Read answer options out loud]

- Yes, I am under treatment/I finished treatment (1)
- No, I have not started treatment yet but I do need treatment (0)
- My tuberculosis test was negative/I do not need treatment (2)
- [Doesn't know/doesn't remember] (88)
- [Doesn't want to answer] (99)

36. In the last 12 months, have you done any heart test?

- Yes (1)
- No (0)
- [Doesn't know/doesn't remember] (88)
- [Doesn't want to answer] (99)

37. I'm now going to ask you some questions about other health and social services.

38. In the last 12 months, did health professionals ask about your personal situation, for example, about your housing situation or your economic/financial situation?

- Yes (1)
- No (0) If this response, jump to 40
- [Doesn't know/doesn't remember] (88) If this response, jump to 40
- [Doesn't want to answer] (99) If this response, jump to 40

39. Did health professionals refer you to any organization that could give you some type of housing or financial support, if you needed help? [INTERVIEWER: Read answer options out loud]

- Yes (1)
- No (0)
- I did not need support (2)
- [Doesn't know/doesn't remember] (88)
- [Doesn't want to answer] (99)

40. In the past 12 months, did health professionals ask about your emotional well-being?

- Yes (1)
- No (0) If this response, jump to 42
- [Doesn't know/doesn't remember] (88) If this response, jump to 42
- [Doesn't want to answer] (99) If this response, jump to 42

41. Did health professionals refer you to a psychologist, psychiatrist, or any support group, if you needed help? [INTERVIEWER: Read answer options out loud]

- Yes (1)
- No (0)
- I did not need support (2)
- [Doesn't know/doesn't remember] (88)
- [Doesn't want to answer] (99)

42. In the past 12 months, did health professionals ask about your use of tobacco, alcohol, or drugs?

- Yes (1)
- No (0) If this response, jump to 44
- [Doesn't know/doesn't remember] (88) If this response, jump to 44
- [Doesn't want to answer] (99) If this response, jump to 44

43. Did health professionals refer you to any specialized services for tobacco, alcohol, or drug use, if you needed help? [INTERVIEWER: Read answer options out loud]

- Yes (1)
- No (0)
- I did not need support (2)
- [Doesn't know/doesn't remember] (88)
- [Doesn't want to answer] (99)

44. When you were diagnosed, did health professionals ask if you needed help telling your sexual partners that you are HIV-positive?

- Yes (1)
- No (0) If this response, jump to 46
- [Doesn't know/doesn't remember] (88) If this response, jump to 46
- [Doesn't want to answer] (99) If this response, jump to 46

45. Did health professionals help you tell your sexual partners that you are HIV-positive?

- Yes (1)
- No (0)
- I did not need help (2)
- [Doesn't know/doesn't remember] (88)
- [Doesn't want to answer] (99)

46. I'm now going to ask you some questions about your HIV treatment.

47. When were you first diagnosed with HIV?

- Less than a year ago (1)
- 1-2 years ago (2)
- 2-5 years ago (3)
- More than 5 years ago (4)
- [Doesn't know/doesn't remember] (88)
- [Doesn't want to answer] (99)

48. After receiving your HIV positive diagnosis, how long did it take until you started treatment?

- I started treatment in less than a month from receiving the diagnosis (1)
- I started treatment between 1 and 3 months from receiving the diagnosis (2)
- I started treatment between 3 and 6 months from receiving the diagnosis (3)
- I started treatment after 6 months from receiving the diagnosis (4)
- [Doesn't know/doesn't remember] (88)
- [Doesn't want to answer] (99)

49. Most HIV medicine has to be taken at specific times, such as "twice a day" or "three times a day." In the LAST 3 DAYS, how often did you take your medications on time? [INTERVIEWER: Read answer options out loud]

- Never (1)
- Sometimes (2)
- Half of the time (3)
- Most of the time (4)
- Always (5)
- I do not need medication (6) If this response, jump to 59
- [Doesn't know/doesn't remember] (88)
- [Doesn't want to answer] (99) If this response, jump to 55

50. Does your HIV/AIDS medication have any special instructions? For example: "take with a meal" or "take with liquids".

- Yes (1)
- No (0) If this response, jump to 52
- [Doesn't know/doesn't remember] (88) If this response, jump to 52
- [Doesn't want to answer] (99) If this response, jump to 52

51. In the LAST 3 DAYS, how often did you follow these special instructions? [INTERVIEWER: Read answer options out loud]

- Never (1)
- Sometimes (2)
- Half of the time (3)
- Most of the time (4)
- Always (5)
- [Doesn't know/doesn't remember] (88)
- [Doesn't want to answer] (99)

52. Some people forget to take their medications over the weekend. DURING THE LAST WEEKEND (on Saturday, Sunday, or both), did you forget to take any of your HIV/AIDS medications?

- Yes (1)
- No (0)
- [Doesn't know/doesn't remember] (88)
- [Doesn't want to answer] (99)

53. When was the last time you forgot to take the medication for your HIV/AIDS treatment? [INTERVIEWER: Read answer options out loud]

- In the past week (1)
- 2 weeks ago (2)
- 3 weeks ago (3)
- 1-3 months ago (4)
- Mora than 3 months ago (5)
- I never forget to take my HIV/AIDS medication (6) If this response, jump to 55
- [Doesn't know/doesn't remember] (88)
- [Doesn't want to answer] (99) If this response, jump to 55

54. Which of the following reasons best describes why you forget to take your HIV/AIDS medication? You can mention more than one reason. [INTERVIEWER: Read answer options out loud]

- Because it is difficult to remember to take so many medications as the doctor orders (1)
- To avoid the side effects of the medications (2)
- Because I think the medications are toxic or cause damage (3)
- Because I did not want other people to see me taking the medication (4)
- Because I was asleep at the time I had to take the medication (5)
- Because I felt sick (6)
- Because I felt depressed or emotionally shaken (7)
- Because I had ran out of medication (8)
- Because I felt good and did not see the need to take the medication (9)
- Because I did not understand the schedule to take the medication (10)
- Because I had not eaten or I had no water at the time I had to take the medication (11)
- Because I had alcohol and/or drugs at the time I had to take the medication (12)
- Because I lost my medication or it was stolen (13)
- Other (14)
- [Doesn't want to answer] (99)

55. The last time that you visited the pharmacy at this health center to get your HIV medication, how long did you have to wait to get your medication? [INTERVIEWER: Read answer options out loud]

- 30 minutes or less (1)
- More than 30 minutes and up to 1 hour (2)
- More than 1 hour and up to 2 hours (3)
- More than 2 hours and up to 3 hours (4)
- More than 3 hours (5)
- [Doesn't know/doesn't remember] (88)
- [Doesn't want to answer] (99)

56. How would you rate the time that it normally takes you to get your HIV medications at this health unit's pharmacy? [INTERVIEWER: Read answer options out loud]

- Very convenient (1)
- Convenient (2)
- More or less convenient (3)
- Inconvenient (4)
- Very inconvenient (5)
- [Doesn't want to answer] (99)

57. The last time you came to the pharmacy at this health center to get your HIV medication, how much medication were you able to get? [INTERVIEWER: Read answer options out loud]

- Medication for less than 1 month (1)
- Medication for 1 month (2)
- Medication for 2 months (3)
- Medication for 3 months (4)
- Medication for 4 months (5)
- Medication for 5 months (6)
- Medication for 6 months (7)
- Medication for more than 6 months (8)
- [Doesn't know/doesn't remember] (88)
- [Doesn't want to answer] (99)

58. How would you rate the amount of HIV medication that you can usually get at this heal unit's pharmacy? [INTERVIEWER: Read answer options out loud]

- Very convenient (1)
- Convenient (2)
- More or less convenient (3)
- Inconvenient (4)
- Very inconvenient (5)
- [Doesn't want to answer] (99)

59. I'm now going to ask you some questions about your satisfaction with the healthcare services at this health unit.

60. In general, how would you rate the quality of healthcare services at this health center? [INTERVIEWER: Read answer options out loud]

- Excellent quality (1)
- Good quality (2)
- Average quality (3)
- Poor quality (4)
- Very bad quality (5)
- [Doesn't want to answer] (99)

61. How would you rate the level of preparation (education and experience) of the health professionals at this health center for the treatment of HIV/AIDS? [INTERVIEWER: Read answer options out loud]

- Excellent quality (1)
- Good quality (2)
- Average quality (3)
- Poor quality (4)
- Very bad quality (5)
- [Doesn't want to answer] (99)

62. How would you rate the quality of communication of the health professionals at this health center with you? [INTERVIEWER: Read answer options out loud]

- Excellent quality (1)
- Good quality (2)
- Average quality (3)
- Poor quality (4)
- Very bad quality (5)
- [Doesn't want to answer] (99)

63. How would you rate the quality of the infrastructure of this health center? [INTERVIEWER: Read answer options out loud]

- Excellent quality (1)
- Good quality (2)
- Average quality (3)
- Poor quality (4)
- Very bad quality (5)
- [Doesn't want to answer] (99)

64. Would you recommend this health center to your HIV positive acquaintances or friends? [INTERVIEWER: Read answer options out loud]

- Definitely yes (1)
- Probably yes (2)
- Probably not (3)
- Definitely not (4)
- I do not know others who are HIV positive (0)
- [Does not want to answer] (99)

65. I'm now going to ask you some questions about yourself.

66. How old are you? [INTERVIEWER: Type "LLL" if patient does not know his/her age or "MMM" if patient does not want to answer]

_____ years old

67. What level of education did you complete? [INTERVIEWER: Read answer options out loud]

- Illiterate (doesn't know how to write/read) (0)
- Incomplete primary education (1)
- Complete primary education (2)
- Incomplete secondary education (3)
- Complete secondary education (4)
- Incomplete tertiary education (8)
- Complete tertiary education (6)
- Post tertiary education/graduate education (7)
- [Doesn't want to answer] (99)

68. What is your race? Please select the race that you identify with the most. [INTERVIEWER: Read answer options out loud]

- Black (1)
- Mulatto (2)
- White (3)
- Asian (4)
- Indigenous (5)
- [Doesn't want to answer] (99)

69.What is your current marital status? [INTERVIEWER: Read answer options out loud]

- Single (never married) (1)
- Married (2)
- Divorced or separated (3)
- Widowed (4)
- Lives with partner but is not married (5)
- [Doesn't want to answer] (99)

70. Why is your gender identity? [INTERVIEWER: Read answer options out loud]

- Woman (1)
- Man (2)
- Travesty (3)
- Female Transsexual (transitioned from male to female) (4)
- Male Transsexual (transitioned from female to male) (5)
- [Doesn't want to answer] (99)

71. What is your sexual orientation? [INTERVIEWER: Read answer options out loud]

- Heterosexual (you feel emotionally and sexually attracted to people of the opposite sex) (1)
- Homosexual (you feel emotionally and sexually attracted to people of the same sex) (2)
- Bisexual (you feel emotionally and sexually attracted to people of both sexes) (3)
- [Doesn't want to answer] (99)

72. What is your current employment status? [INTERVIEWER: Read answer options out loud]

- Employed (1)
- Self-employed (2)
- Unemployed with no financial support/aid (3) If this response, jump to 74
- Unemployed with some financial support/aid (4)
- Retired (5)
- [Doesn't want to answer] (99)

73. What is your monthly income? [INTERVIEWER: If the patient is self-employed, please ask for last month's income. Enter 'LLL' if patient doesn't know and 'MMM' if patient doesn't want to answer]

____

74. What is your full name, as it appears on your RG? [INTERVIEWER: Write down ALL the names and surnames without accents, for example: JULIA ISABEL DA SILVA MADEIRA. Enter "MMM" if the patient does not want to provide his/her name]

____

75. What is your date of birth? [INTERVIEWER: Enter "11 de Janeiro de 2016" if patient does not want to provide his/her date of birth]

____

76. In what city were you born? [INTERVIEWER: Write down the name of the city without accents, for example: SAO PAULO. Enter 'LLL' if patient doesn't know and 'MMM' if patient doesn't want to answer]

____

77. Do you currently live in Manaus?

- Yes (1)
- No (0)
- [Doesn't want to answer] (99)

78. What is the name of your mother, as registered in your RG? [INTERVIEWER: Write down ALL the names and surnames without accents, for example: JULIA ISABEL DA SILVA MADEIRA. Enter "MMM" if the patient does not want to provide his/her mother's name]

____

79. END OF THE INTERVIEW: We have finished the interview. Many thanks for your answers. This information will be very useful to improve the quality of services at this health unit.

80. [INTERVIEWER: Save and submit the questionnaire]
