# Supplementary material for: HIV/AIDS health services in Manaus, Brazil: patient perception of quality and its influence on adherence to antiretroviral treatment
Source: BMC Health Serv Res. 2019 May 30;19:344. doi: 10.1186/s12913-019-4062-9 (PMC6543648; doi:10.1186/s12913-019-4062-9)
Supplement: Supplementary file 3 — General Factors Associated with Patient Satisfaction. This file presents regression results on factors associated with patient satisfaction at both the central hospital and the decentralized health units. (PDF 57 kb) [file 12913_2019_4062_MOESM3_ESM.pdf]

## Factors Associated with Highest Patient Satisfaction (Central Hospital and Decentralized Health Units)

Weighted analysis using sampling weights to reflect the real distribution of patient population across health centers.

| | Univariable Analysis N=812 | | | | | Multivariable Analysis N=803 | | | | | Multivariable Analysis N=810 | | | | |
|---|---|---|---|---|---|---|---|---|---|---|---|---|---|---|---|
| | Analysis of individual factors | | | | | Full model including ALL patient characteristics and ALL health center characteristics | | | | | Final model including patient characteristics and health center characteristics that best explain highest patient satisfaction | | | | |
| | Crude Odd Ratios | 95% CI | | p-value | | Adjusted Odd Ratios | 95% CI | | p-value | | Adjusted Odd Ratios | 95% CI | | p-value | |
| ***Patient's characteristics*** | | | | | | | | | | | | | | | |
| **Age group** | | | | | | | | | | | | | | | |
| 18-25 years old | 1 | | | | | 1 | | | | | | | | | |
| 26-35 years old | 1.03 | 0.66 | 1.59 | 0.883 | | 1.14 | 0.44 | 2.92 | 0.689 | | | | | | |
| 36-45 years old | 1.11 | 0.78 | 1.58 | 0.489 | | 1.42 | 0.42 | 4.82 | 0.424 | | | | | | |
| 46-55 years old | 1.12 | 0.75 | 1.69 | 0.492 | | 1.47 | 0.48 | 4.49 | 0.354 | | | | | | |
| More than 55 years old | 0.97 | 0.94 | 1.01 | 0.102 | | 1.34 | 0.39 | 4.61 | 0.501 | | | | | | |
| **Gender** (1) | | | | | | | | | | | | | | | |
| Women | 1 | | | | | 1 | | | | | | | | | |
| Men | 1.06 | 0.91 | 1.23 | 0.319 | | 0.74 | 0.62 | 0.89 | 0.013 | ** | | | | | |
| **Education** | | | | | | | | | | | | | | | |
| Illiterate or incomplete primary education | 1 | | | | | 1 | | | | | 1 | | | | |
| Complete primary education | 1.90 | 1.78 | 2.02 | 0.000 | *** | 1.57 | 0.68 | 3.60 | 0.184 | | 1.90 | 1.15 | 3.14 | 0.021 | ** |
| Complete secondary education | 1.54 | 0.86 | 2.74 | 0.114 | | 1.17 | 0.55 | 2.53 | 0.552 | | 1.39 | 0.81 | 2.40 | 0.176 | ** |
| Complete tertiary education or higher | 2.41 | 0.95 | 6.09 | 0.059 | * | 1.68 | 0.94 | 2.99 | 0.065 | * | 2.16 | 0.95 | 4.91 | 0.061 | * |
| **Race** (2) | | | | | | | | | | | | | | | |
| Black | 1 | | | | | 1 | | | | | | | | | |
| Mulatto | 1.33 | 1.23 | 1.42 | 0.000 | *** | 0.91 | 0.54 | 1.52 | 0.595 | | | | | | |
| White | 1.17 | 0.85 | 1.62 | 0.261 | | 0.66 | 0.40 | 1.10 | 0.080 | * | | | | | |
| Asian | 2.99 | 1.06 | 8.46 | 0.043 | ** | 1.88 | 0.98 | 3.59 | 0.053 | * | | | | | |
| Indigenous | 0.18 | 0.03 | 0.90 | 0.041 | ** | 0.12 | 0.00 | 6.69 | 0.194 | | | | | | |
| **Sexual orientation** (3) | | | | | | | | | | | | | | | |
| Heterosexual | 1 | | | | | 1 | | | | | | | | | |
| Homosexual | 1.52 | 1.02 | 2.25 | 0.042 | ** | 1.56 | 1.21 | 2.01 | 0.011 | ** | | | | | |
| Bisexual | 1.35 | 0.79 | 2.32 | 0.213 | | 1.58 | 1.02 | 2.45 | 0.045 | ** | | | | | |
| **Monthly Income** | | | | | | | | | | | | | | | |
| No income | 1 | | | | | 1 | | | | | | | | | |
| R$500 or less | 0.93 | 0.58 | 1.50 | 0.721 | | 0.75 | 0.37 | 1.50 | 0.275 | | | | | | |
| More than R$ 500 and up to R$ 1,000 | 0.75 | 0.46 | 1.20 | 0.177 | | 0.76 | 0.43 | 1.36 | 0.235 | | | | | | |
| More than R$ 1,000 and up to R$ 2,000 | 1.34 | 0.56 | 3.19 | 0.425 | | 1.08 | 0.24 | 4.95 | 0.876 | | | | | | |
| More than R$ 2,000 | 1.28 | 0.63 | 2.62 | 0.414 | | 0.82 | 0.71 | 0.95 | 0.023 | ** | | | | | |
| **Place of residence** | | | | | | | | | | | | | | | |
| Manaus | 1 | | | | | 1 | | | | | 1 | | | | |
| Outside of Manaus | 1.09 | 0.68 | 1.73 | 0.669 | | 2.06 | 1.84 | 2.30 | 0.000 | *** | 1.68 | 1.56 | 1.81 | 0.000 | *** |
| ***Health center characteristics*** | | | | | | | | | | | | | | | |
| **Commute time to health center** (4) | | | | | | | | | | | | | | | |
| Less than 30 minutes | 1 | | | | | 1 | | | | | | | | | |
| 30 minutes to 1 hour | 0.53 | 0.28 | 0.99 | 0.048 | ** | 0.65 | 0.25 | 1.69 | 0.247 | | | | | | |
| More than 1 hour | 0.39 | 0.16 | 0.94 | 0.040 | ** | 0.60 | 0.21 | 1.69 | 0.212 | | | | | | |
| **Convinience of health center's location** | | | | | | | | | | | | | | | |
| Inconvenient/Very inconvenient | 1 | | | | | 1 | | | | | 1 | | | | |
| More or less | 0.51 | 0.29 | 0.90 | 0.029 | ** | 0.43 | 0.31 | 0.59 | 0.004 | *** | 0.46 | 0.35 | 0.60 | 0.001 | *** |
| Convenient/Very convenient | 3.00 | 2.44 | 3.69 | 0.000 | *** | 2.38 | 1.38 | 4.10 | 0.015 | ** | 2.70 | 2.18 | 3.35 | 0.000 | *** |
| **Waiting time** (5) | | | | | | | | | | | | | | | |
| Less than 30 minutes | 1 | | | | | 1 | | | | | 1 | | | | |
| 30 minutes to 1 hour | 0.52 | 0.11 | 2.44 | 0.325 | | 0.68 | 0.08 | 6.18 | 0.620 | | 0.58 | 0.12 | 2.83 | 0.413 | |
| 1-2 hours | 0.42 | 0.12 | 1.46 | 0.133 | | 0.65 | 0.09 | 4.75 | 0.542 | | 0.52 | 0.13 | 2.11 | 0.282 | |
| 2-3 hours | 0.33 | 0.08 | 1.41 | 0.108 | | 0.49 | 0.05 | 4.68 | 0.389 | | 0.45 | 0.08 | 2.46 | 0.279 | |
| More than 3 hours | 0.31 | 0.26 | 0.37 | 0.000 | *** | 0.49 | 0.24 | 1.00 | 0.050 | ** | 0.40 | 0.31 | 0.51 | 0.000 | *** |
| **Time to reschedule a missed appointment** | | | | | | | | | | | | | | | |
| A week (7 days) or less | 1 | | | | | 1 | | | | | | | | | |
| Between 1 week and 1 month (30 days) | 0.52 | 0.46 | 0.59 | 0.000 | *** | 0.80 | 0.56 | 1.15 | 0.144 | | | | | | |
| More than 1 month | 0.52 | 0.44 | 0.60 | 0.000 | *** | 0.83 | 0.34 | 2.05 | 0.557 | | | | | | |
| **Respectful treatment from nurses** | | | | | | | | | | | | | | | |
| No | 1 | | | | | 1 | | | | | 1 | | | | |
| Yes | 1.47 | 1.20 | 1.79 | 0.004 | *** | 1.40 | 0.99 | 1.97 | 0.055 | * | 1.50 | 1.03 | 2.20 | 0.040 | ** |
| **Respectful treatment from doctors** | | | | | | | | | | | | | | | |
| No | 1 | | | | | 1 | | | | | | | | | |
| Yes | 4.74 | 0.75 | 29.97 | 0.082 | * | 3.74 | 0.52 | 26.70 | 0.123 | | | | | | |
| **Health Center** | | | | | | | | | | | | | | | |
| FMT | 1 | | | | | 1 | | | | | 1 | | | | |
| SAE 1 | 1.43 | 0.69 | 2.97 | 0.264 | | 1.14 | 0.42 | 3.12 | 0.706 | | 1.20 | 0.64 | 2.25 | 0.485 | |
| SAE 2 | 5.02 | 2.47 | 10.21 | 0.002 | *** | 3.23 | 0.75 | 13.89 | 0.083 | * | 3.34 | 1.42 | 7.90 | 0.015 | ** |
| SAE 3 | 3.38 | 3.02 | 3.79 | 0.000 | *** | 2.36 | 1.79 | 3.11 | 0.002 | *** | 2.52 | 2.24 | 2.85 | 0.000 | *** |
| SAE 4 | 1.85 | 1.23 | 2.79 | 0.012 | ** | 1.02 | 0.15 | 6.80 | 0.973 | | 1.03 | 0.41 | 2.59 | 0.941 | |

*** p<0.01, ** p<0.05, * p<0.1

(1) 3 patients did not want to disclose their gender
(2) 2 patients did not report their race
(3) 1 patient did not disclose sexual orientation
(4) 1 patient did not respond the question
(5) 2 patients did not remember the wait time from last visit
