# Supplementary material for: HIV/AIDS health services in Manaus, Brazil: patient perception of quality and its influence on adherence to antiretroviral treatment
Source: BMC Health Serv Res. 2019 May 30;19:344. doi: 10.1186/s12913-019-4062-9 (PMC6543648; doi:10.1186/s12913-019-4062-9)
Supplement: Supplementary file 4 — Factors Associated with Patient Satisfaction at Central Hospital. This file presents regression results on factors associated with patient satisfaction at the central hospital. (PDF 55 kb) [file 12913_2019_4062_MOESM4_ESM.pdf]

**Factors Associated with Highest Patient Satisfaction at Central Hospital**	**Univariable Analysis N=410**					**Multivariable Analysis N=395**					**Multivariable Analysis N=409**				
	Analysis of individual factors					Full model including ALL patient characteristics and ALL health center characteristics					Final model including patient characteristics and health center characteristics that best explain highest patient satisfaction				
	Crude Odd Ratios	95% CI		p-value		Adjusted Odd Ratios	95% CI		p-value		Adjusted Odd Ratios	95% CI		p-value	
Patient's characteristics															
**Age group**															
18-25 years old	1					1									
26-35 years old	1.29	0.50	3.32	0.592		1.69	0.59	4.87	0.332						
36-45 years old	1.57	0.64	3.83	0.326		2.04	0.72	5.78	0.179						
46-55 years old	1.62	0.61	4.28	0.334		2.27	0.72	7.15	0.162						
More than 55 years old	1.41	0.45	4.47	0.555		1.95	0.48	7.93	0.350						
**Gender** (1)															
Women	1					1									
Men	0.91	0.55	1.50	0.717		0.73	0.38	1.41	0.344						
**Education**															
Illiterate or incomplete primary education	1					1									
Complete primary education	1.89	0.86	4.16	0.112		1.43	0.58	3.54	0.435						
Complete secondary education	1.32	0.69	2.55	0.405		0.97	0.45	2.09	0.936						
Complete tertiary education or higher	2.09	0.91	4.84	0.084	*	1.50	0.51	4.44	0.461						
**Race**															
Black	1					1									
Mulatto	1.29	0.43	3.92	0.648		0.79	0.23	2.69	0.709						
White	0.93	0.25	3.46	0.914		0.50	0.12	2.12	0.344						
Asian	3.00	0.50	17.95	0.229		1.55	0.21	11.58	0.671						
Indigenous	1	(omitted)				-	(omitted)								
**Sexual orientation** (2)															
Heterosexual	1					1									
Homosexual	1.27	0.70	2.30	0.426		1.41	0.64	3.11	0.392						
Bisexual	1.22	0.53	2.81	0.645		1.78	0.62	5.13	0.283						
**Monthly Income**															
No income	1					1					1				
R$500 or less	0.59	0.19	1.89	0.377		0.95	0.22	4.16	0.947		0.43	0.13	1.44	0.169	
More than R$ 500 and up to R$ 1,000	0.66	0.35	1.25	0.205		1.18	0.40	3.46	0.761		0.54	0.27	1.06	0.072	*
More than R$ 1,000 and up to R$ 2,000	1.17	0.60	2.30	0.649		1.73	0.60	4.98	0.309		0.87	0.42	1.82	0.719	
More than R$ 2,000	0.92	0.37	2.27	0.857		-	(omitted)				0.59	0.22	1.59	0.299	
**Place of residence**															
Manaus	1					1					1				
Outside of Manaus	1.23	0.65	2.33	0.517		2.01	0.93	4.37	0.078	*	1.98	0.96	4.09	0.064	*
Health center characteristics															
**Commute time to health center** (3)															
Less than 30 minutes	1					1					1				
30 minutes to 1 hour	0.50	0.26	0.96	0.038	**	0.55	0.26	1.16	0.118		0.55	0.28	1.12	0.099	*
More than 1 hour	0.42	0.22	0.77	0.005	***	0.48	0.23	1.02	0.056	*	0.48	0.23	0.98	0.044	**
**Convinience of health center's location**															
Inconvenient/Very inconvenient	1					1					1				
More or less	0.45	0.13	1.56	0.208		0.40	0.11	1.46	0.164		0.44	0.12	1.58	0.210	
Convenient/Very convenient	2.58	1.17	5.66	0.018	**	2.37	1.00	5.61	0.050	*	2.49	1.07	5.77	0.034	**
**Waiting time**															
Less than 30 minutes	1					1					1				
30 minutes to 1 hour	0.61	0.25	1.48	0.276		0.89	0.32	2.48	0.816		0.80	0.30	2.13	0.651	
1-2 hours	0.57	0.23	1.39	0.215		0.90	0.32	2.53	0.843		0.80	0.30	2.13	0.650	
2-3 hours	0.45	0.18	1.16	0.098	*	0.59	0.20	1.76	0.347		0.59	0.21	1.66	0.318	
More than 3 hours	0.46	0.18	1.15	0.097	*	0.71	0.25	2.06	0.531		0.65	0.23	1.79	0.400	
**Time to reschedule a missed appointment**															
A week (7 days) or less	1					1									
Between 1 week and 1 month (30 days)	2.44	0.29	20.18	0.408		1.85	0.20	17.13	0.590						
More than 1 month	2.48	0.31	19.75	0.391		1.84	0.20	16.62	0.586						
**Respectful treatment from nurses**															
No	1					1					1				
Yes	1.30	0.70	2.40	0.409		1.29	0.66	2.54	0.461		1.26	0.66	2.43	0.483	
**Respectful treatment from doctors**															
No	1					1					1				
Yes	5.10	0.67	38.56	0.115		4.60	0.55	38.10	0.157		5.23	0.66	41.67	0.118	

*** p<0.01, ** p<0.05, * p<0.1

(1) 2 patients did not disclose gender
(2) 1 patient did not disclose sexual orientation
(3) 1 patient did not answer the question
