# Supplementary material for: HIV/AIDS health services in Manaus, Brazil: patient perception of quality and its influence on adherence to antiretroviral treatment
Source: BMC Health Serv Res. 2019 May 30;19:344. doi: 10.1186/s12913-019-4062-9 (PMC6543648; doi:10.1186/s12913-019-4062-9)
Supplement: Supplementary file 5 — Factors Associated with Patient Satisfaction at Decentralized Health Units. This file presents regression results on factors associated with patient satisfaction at the decentralized health units. (PDF 57 kb) [file 12913_2019_4062_MOESM5_ESM.pdf]

Factors Associated with Highest Patient Satisfaction at Decentralized Health Units

Weighted analysis using sampling weights to reflect the real distribution of patient population across SAEs

	Univariable Analysis N=402					Multivariable Analysis N=397					Multivariable Analysis N=400				
	Analysis of individual factors					Full model including ALL patient characteristics and ALL health center characteristics					Final model including patient characteristics and health center characteristics that best explain highest patient satisfaction				
	Crude Odd Ratios	95% CI		p-value		Adjusted Odd Ratios	95% CI		p-value		Adjusted Odd Ratios	95% CI		p-value	
Patient's characteristics															
**Age group**															
18-25 years old	1					1									
26-35 years old	0.89	0.56	1.40	0.516		0.79	0.74	0.83	0.001	***					
36-45 years old	1.07	0.50	2.31	0.818		0.68	0.43	1.08	0.077	*					
46-55 years old	0.98	0.56	1.71	0.928		1.24	0.56	2.75	0.458						
More than 55 years old	0.93	0.30	2.86	0.872		1.71	0.20	14.76	0.488						
**Gender** (1)															
Women	1					1									
Men	1.71	1.56	1.86	0.000	***	0.62	0.47	0.83	0.013	**					
**Education**															
Illiterate or incomplete primary education	1					1					1				
Complete primary education	1.79	1.74	1.84	0.000	***	3.54	1.01	12.38	0.049	**	2.66	1.60	4.42	0.006	***
Complete secondary education	2.50	1.52	4.11	0.007	***	3.06	1.37	6.81	0.021	**	2.41	1.24	4.69	0.021	**
Complete tertiary education or higher	3.71	1.86	7.39	0.006	***	2.27	1.41	3.65	0.012	**	2.13	1.22	3.69	0.019	**
**Race** (2)															
Black	1					1									
Mulatto	1.42	0.43	4.66	0.460		1.00	0.40	2.52	0.998						
White	2.08	0.47	9.21	0.245		1.08	0.77	1.51	0.535						
Asian	4.30	0.37	49.75	0.173		1.79	0.47	6.75	0.258						
Indigenous	1.25	0.89	1.75	0.138		0.50	0.08	3.13	0.316						
**Sexual orientation**															
Heterosexual	1					1					1				
Homosexual	1.96	1.46	2.64	0.003	***	2.96	1.53	5.75	0.014	**	2.26	1.56	3.26	0.004	***
Bisexual	1.38	0.97	1.98	0.066	*	1.73	1.48	2.02	0.001	***	1.23	0.83	1.81	0.215	
**Monthly Income**															
No income	1					1					1				
R$500 or less	2.25	1.45	3.50	0.007	***	2.95	1.99	4.38	0.003	***	2.52	1.64	3.87	0.004	***
More than R$ 500 and up to R$ 1,000	1.94	1.54	2.44	0.001	***	1.87	1.14	3.06	0.028	**	1.65	0.94	2.92	0.070	*
More than R$ 1,000 and up to R$ 2,000	2.63	1.80	3.83	0.002	***	2.64	1.89	3.67	0.003	***	2.35	2.03	2.72	0.000	***
More than R$ 2,000	5.71	5.37	6.08	0.000	***	6.67	3.76	11.83	0.002	***	5.52	3.70	8.26	0.000	***
**Place of residence**															
Manaus	1					1									
Outside of Manaus	1.32	0.51	3.44	0.460		2.67	1.10	6.48	0.038	**					
Health center characteristics															
**Commute time to health center**															
Less than 30 minutes	1					1									
30 minutes to 1 hour	0.70	0.48	1.04	0.066	*	1.07	0.51	2.24	0.791						
More than 1 hour	0.45	0.24	0.86	0.026	**	0.74	0.40	1.36	0.212						
**Convinience of health center's location**															
Inconvenient/Very inconvenient	1					1									
More or less	1.86	0.11	32.12	0.578		1.00	0.07	15.02	0.997						
Convenient/Very convenient	9.05	3.18	25.78	0.004	***	5.26	1.08	25.68	0.045	**					
**Waiting time** (3)															
Less than 30 minutes	1					1					1				
30 minutes to 1 hour	0.52	0.31	0.86	0.022	**	0.51	0.23	1.10	0.068	*	0.51	0.29	0.92	0.033	**
1-2 hours	0.30	0.19	0.46	0.001	***	0.23	0.10	0.53	0.011	**	0.24	0.18	0.32	0.000	***
2-3 hours	0.33	0.24	0.45	0.001	***	0.29	0.15	0.59	0.011	**	0.32	0.21	0.50	0.002	***
More than 3 hours	0.08	0.06	0.12	0.000	***	0.07	0.06	0.07	0.000	***	0.08	0.05	0.12	0.000	***
**Time to reschedule a missed appointment**															
A week (7 days) or less	1					1									
Between 1 week and 1 month (30 days)	0.46	0.29	0.75	0.012	**	0.49	0.46	0.52	0.000	***					
More than 1 month	0.70	0.58	0.85	0.007	***	0.60	0.17	2.08	0.285						
**Respectful treatment from nurses**															
No	1					1					1				
Yes	1.73	1.28	2.32	0.007	***	3.18	0.92	10.95	0.059	*	3.64	2.48	5.35	0.001	***
**Respectful treatment from doctors**															
No	1					1									
Yes	2.00	1.18	3.38	0.022	**	1.53	0.60	3.92	0.244						
**SAE**															
SAE 1	1					1					1				
SAE 2	3.51	3.19	3.87	0.000	***	1.98	1.31	2.99	0.014	**	2.48	1.84	3.33	0.001	***
SAE 3	2.37	1.19	4.70	0.025	**	1.20	0.80	1.79	0.247		1.60	0.96	2.67	0.063	*
SAE 4	1.29	0.91	1.85	0.115		0.30	0.27	0.33	0.000	***	0.53	0.46	0.60	0.000	***

*** p<0.01, ** p<0.05, * p<0.1

(1) 1 patient did not disclose gender
(2) 2 patients did not report race
(3) 2 patients did not remember the waiting time from last visit
