# Supplementary material for: HIV/AIDS health services in Manaus, Brazil: patient perception of quality and its influence on adherence to antiretroviral treatment
Source: BMC Health Serv Res. 2019 May 30;19:344. doi: 10.1186/s12913-019-4062-9 (PMC6543648; doi:10.1186/s12913-019-4062-9)
Supplement: Supplementary file 6 — Effect of Patient Satisfaction on Adherence to ART. This file presents regression results on the effect of patient satisfaction on adherence to treatment at both the central hospital and the decentralized health units. (PDF 56 kb) [file 12913_2019_4062_MOESM6_ESM.pdf]

**Effect of Patient Satisfaction on Adherence to ART (Central Hospital and Decentralized Health Units)**	**Univariable Analysis N=502 (A)**					**Multivariable Analysis Effect of Overall Satisfaction N=499**				
Adherence to ART is measured through viral load. Viral load less than 1,000 copies/mL indicates patient adheres to ART. Sample only includes patients with at least one year of exposure to ART.	Analysis of individual factors					Multi-level model including all patient and health center characteristics as control variables				
	Crude Odd Ratios	95% CI		p-value		Adjusted Odd Ratios	95% CI		p-value	
**Overall Satisfaction with health center (A)**										
Very satisfied	1.83	1.02	3.29	0.042	**	1.99	1.02	3.89	0.043	**
Otherwise	1					1				
Patient's characteristics										
**Age group**										
18-25 years old	1					1				
26-35 years old	1.42	0.72	2.77	0.309		2.02	0.93	4.35	0.074	*
36-45 years old	1.70	0.86	3.34	0.125		3.28	1.39	7.73	0.007	***
46-55 years old	3.18	1.29	7.86	0.012	**	5.69	1.95	16.62	0.001	***
More than 55 years old	5.77	1.26	26.48	0.024	**	11.91	2.07	68.50	0.006	***
**Gender** (1)										
Women	1					1				
Men	1.17	0.73	1.89	0.513		0.82	0.44	1.53	0.537	
**Education**										
Primary education or less	1					1				
Secondary education or higher	1.09	0.67	1.76	0.734		0.90	0.51	1.60	0.729	
**Race** (2)										
Black	1					1				
Mulatto	1.05	0.39	2.86	0.923		1.02	0.34	3.00	0.977	
White	1.86	0.54	6.47	0.327		1.82	0.47	6.94	0.383	
Asian	0.65	0.10	4.23	0.654		0.89	0.12	6.79	0.907	
Indigenous	0.49	0.11	2.25	0.358		0.49	0.09	2.63	0.404	
**Sexual orientation**										
Heterosexual	1					1				
Otherwise	1.45	0.86	2.46	0.167		1.99	0.97	4.08	0.061	*
**Employment**										
Employed	1					1				
Self-employed	1.01	0.47	2.17	0.986		0.85	0.35	2.11	0.732	
Unemployed without income	0.50	0.28	0.89	0.020	**	0.63	0.22	1.87	0.409	
Unemployed with financial support	0.53	0.26	1.07	0.076	*	0.69	0.29	1.64	0.396	
Retired	2.69	0.61	11.93	0.192		2.30	0.45	11.84	0.318	
**Monthly Income**										
No income	1					1				
R$500 or less	2.43	0.95	6.22	0.064	*	1.71	0.42	6.94	0.451	
More than R$ 500 and up to R$ 1,000	1.41	0.79	2.50	0.245		0.78	0.26	2.32	0.652	
More than R$ 1,000 and up to R$ 2,000	2.36	1.17	4.78	0.017	**	1.30	0.44	3.90	0.634	
More than R$ 2,000	2.33	0.91	5.97	0.079	*	(omitted)				
**Place of residence**										
Manaus	1					1				
Outside of Manaus	0.92	0.46	1.85	0.82		1.04	0.45	2.42	0.926	
Health center characteristics										
**Commute time to health center** (3)										
Less than 30 minutes	1					1				
30 minutes to 1 hour	0.88	0.48	1.64	0.693		0.90	0.45	1.80	0.771	
More than 1 hour	0.90	0.49	1.65	0.743		1.00	0.48	2.08	0.990	
**Convinience of health center's location**										
Inconvenient/Very inconvenient	1					1				
More or less	1.27	0.47	3.43	0.644		1.33	0.45	3.93	0.611	
Convenient/Very convenient	0.85	0.40	1.82	0.684		0.55	0.23	1.32	0.182	
**Waiting time**										
Less than 30 minutes	1					1				
30 minutes to 1 hour	1.33	0.62	2.85	0.458		1.76	0.75	4.16	0.195	
1-3 hours	1.02	0.49	2.11	0.964		1.63	0.70	3.82	0.257	
More than 3 hours	1.02	0.44	2.36	0.959		1.77	0.67	4.67	0.251	
**Time to reschedule a missed appointment**										
A week (7 days) or less	1					1				
Between 1 week and 1 month (30 days)	0.51	0.24	1.11	0.091	*	0.64	0.26	1.58	0.337	
More than 1 month	0.77	0.37	1.62	0.491		1.40	0.53	3.74	0.499	
**Respectful treatment from health professionals**										
No	1					1				
Yes	1.46	0.85	2.49	0.169		1.30	0.71	2.38	0.397	
**Health Center**										
FMT	1					1				
SAEs	1.77	1.07	2.92	0.025	**	0.26	0.02	3.22		

*** p<0.01, ** p<0.05, * p<0.1
Multi-variate model: LR test vs. logistic model: chibar2(01) = 4.12 Prob >= chibar2 = 0.0212
(A) There was information on last viral load test for 502 patients who had been exposed to ART for at least one year

(1) 1 patient did not disclose gender
(2) 1 patient did not report race
(3) 1 patient did not respond the question
